# Comparison of Occupational Performance in Immersive Virtual and Real Environments Among Patients With Stroke: Observational Randomized Crossover Pilot Study

**DOI:** 10.2196/58388

**Published:** 2024-11-15

**Authors:** Xijun Wei, Ping Zhou, Yixi Wei, Dashuang Wu, Ping Qin, Yingying Zhang, Jing Zhu, Zhanbing Ren, Hai Li, Yumei Zhang

**Affiliations:** 1 Department of Rehabilitation Medicine, Beijing Tiantan Hospital, Capital Medical University, Beijing, China; 2 Department of Rehabilitation Medicine, Shenzhen Hospital, Southern Medical University, Shenzhen, China; 3 Department of Occupational Therapy, School of Rehabilitation Sciences, Southern Medical University, Shenzhen, China; 4 Rehabilitation Lab of Mix Reality, Shenzhen Hospital, Southern Medical University, Shenzhen, China; 5 Department of Gynecology, Shenzhen Hospital, Southern Medical University, Shenzhen, China; 6 Department of Rehabilitation Sciences, Faculty of Health and Social Sciences, The Hong Kong Polytechnic University, Kowloon, China (Hong Kong); 7 College of Physical Education, Shenzhen University, Shenzhen, China

**Keywords:** instrumental activities of daily living, immersive virtual reality, occupational performance, stroke rehabilitation, occupational therapy

## Abstract

**Background:**

Conventional rehabilitation approaches involve therapists simulating various occupational tasks in health care settings or recreating real-life situations to assess and train patients in instrumental activities of daily living (IADLs). As an alternative, immersive virtual reality (IVR) has been widely used in stroke rehabilitation for years, but research comparing occupational performance between virtual and real environments is limited.

**Objective:**

This study aims to introduce a novel IVR shopping system designed for patients with stroke and to investigate the correlation of occupational performance in virtual and real environments among patients with stroke.

**Methods:**

Ten patients with stroke were recruited from the Department of Rehabilitation Medicine, Shenzhen Hospital, Southern Medical University, who met the inclusion and exclusion criteria for this observational, randomized crossover study; the patients were predominantly male (n=7), had experienced ischemic stroke (n=9), were aged 14 to 73 years, and had a time since stroke of 1 to 42 months. All patients attempted shopping tasks in virtual and real environments. The Mini-Mental State Examination (MMSE), Timed Up and Go Test (TUGT), modified Barthel index (MBI), and Lawton index (LI) were used to assess cognition, ambulation, and activities of daily living. Memory capacity and duration in the virtual and real environments were recorded as the primary parameters of occupational performance. The Wilcoxon test and Spearman correlation coefficients were used to analyze the differences and correlations between the 2 environments.

**Results:**

The Wilcoxon test showed no significant differences between the virtual and real environments in memory capacity and duration of task completion (*P*>.99 and *P*=.99), and memory capacity in both environments correlated with the LI (ρ=0.81; *P*=.005). Memory duration had a relationship with the TUGT in the virtual environment (ρ=0.68; *P*=.03) and a borderline negative correlation with MMSE in the real environment (ρ=−0.58; *P*=.08).

**Conclusions:**

Considering the small sample size used in this study and the study’s limitations, despite the significant correlation between shopping performance in IVR and the real world, it is still too early to conclude that IVR is a noninferior approach, but it presents the potential to be an alternative for assessment and training in IADLs when resources are limited. However, further research is needed to investigate the psychometric properties, clinical effects, and impact of virtual training on real-world performance. The implications for practice might include the following: (1) occupational performance in virtual shopping might be the same as real-world shopping, and more virtual IADLs could thus be developed; (2) virtual IADL assessment and training systems could be used in remote locations or locations with limited resources; and (3) more objective parameters of IADLs could be extracted from virtual environments.

## Introduction

Based on the latest statistics, China is facing the greatest challenge worldwide related to stroke [1]. Many patients with stroke experience long-term disability in the somatosensory system, cognition, activities of daily living, and their vocation, which could significantly impact their quality of life [2]. Stroke rehabilitation involves addressing body function, activities, participation, and environmental and personal factors based on the framework of the *International Classification of Functioning, Disability and Health* (*ICF*) [3]. A crucial *ICF* domain is instrumental activities of daily living (IADLs), which occupational therapists assess through observation in real environments or questionnaires such as the Functional Activities Questionnaire and Lawton index (LI) [4-6]. However, it is challenging to achieve consistency between subjective questionnaires and observations. Additionally, some IADL training content, such as shopping, public transportation, and spatial orientation in the community, is difficult to implement in general hospitals and rehabilitation settings due to limited human resources and instruments. Occasionally, patients can access nearby real environments for assessment and training under the supervision of occupational therapists.

For patients with stroke, occupational performance in IADLs such as shopping, taking public transportation, and financial management can be challenging. These occupational activities require a combination of motor and cognitive skills, which may be affected by stroke. As a result, stroke rehabilitation is essential to help patients regain function and independence. Virtual reality (VR) technology has emerged as a potential tool for stroke rehabilitation [7]. It can provide a controlled, safe environment for patients to practice IADLs, with less resource consumption and fewer of the risks associated with real-world activities. Previous studies have shown that VR can be effective in the rehabilitation of the upper extremities [8], lower extremities and balance [9], and cognition [10], as well as in psychological rehabilitation [11]. However, most studies used commercial VR games or intensive, repetitive exercises to train body functions. Although current evidence suggests that VR-based rehabilitation is beneficial [12,13], there are still challenges in assessing and training patients in the *activity* domain of the *ICF*, which includes IADLs. Son and Park [14] found that cognitive training based on VR could benefit patients with mild cognitive impairment (MCI) and Alzheimer disease in IADLs. Other studies have also shown that immersive VR (IVR) and serious games provide more benefits for patients with stroke in upper extremity recovery compared with nonimmersive VR and commercial VR games [8,15]. This is because these technologies use varied human-computer interactions, allowing patients to engage more fully with the virtual environment. As such, IVR and serious games hold promise for stroke rehabilitation, providing an alternative approach to traditional methods.

Compared with nonimmersive VR, IVR has more freedom in system design for human-computer interactions. This allows for a more natural simulation of movement patterns during IADL tasks such as turning the head toward lateral or rear targets or moving within a large space. Conversely, nonimmersive VR requires the user to continuously watch the front screen during interactions [15]. Palacios-Navarro and Hogan [16] identified advantages of IVR for gait, balance, and upper extremity rehabilitation, but their interventions were not specifically related to IADLs. Compared with intensive and repetitive training, distributed and IADL-related training significantly differ in user experience, and occupational performance in IVR may differ from previous studies. As such, further research is needed to explore the potential benefits of IVR for stroke rehabilitation, particularly in IADL tasks.

Well-designed human-computer interactions can prompt patients to initiate activities, but feedback in virtual environments is currently limited, with most systems only simulating auditory, visual, and vibration senses. Somatosensory simulation remains a challenge [7]. Therefore, it is meaningful to explore the value of activities implemented in IVR in comparison to real-world activities, as this technology could serve as an alternative approach [17]. Previous research has investigated movement kinematic and postural control differences between virtual and real golf putting in undergraduate students. The results showed that haptic conditions improved swing kinematics compared to pure VR, and movement patterns in the virtual environment were closer to those in the real environment [18]. These findings are partially supported by a study conducted by Ferroni and colleagues [19], which showed that remapping of peripersonal space can occur in the real world with somatosensory input but not in a virtual environment without somatosensory feedback. However, a review conducted by Tuena and colleagues [20] concluded that nonimmersive virtual environments are suitable for spatial memory in people with MCI, and there have been limited studies exploring occupational performance in IVR. IADLs require multidimensional functions like cognition, motor skills, and environmental interaction. Therefore, IADL performance in IVR may differ from real-world performance.

Currently, there is a lack of studies comparing occupational performance in IVR and real environments when patients who have had a stroke engage in IADL tasks [21]. Shopping is the most complex activity among IADLs and is difficult to carry out in the real world. Virtual shopping is attracting the interest of rehabilitation professionals [22,23]. Therefore, this study aimed to investigate the correlation between the occupational performance of patients with stroke in virtual and real shopping tasks and to investigate the possibility of virtual shopping as an alternative approach for IADL assessment and training. We hypothesized that patients would perform similarly in the IVR and real environments.

## Methods

### Study Design

**Figure 1.**
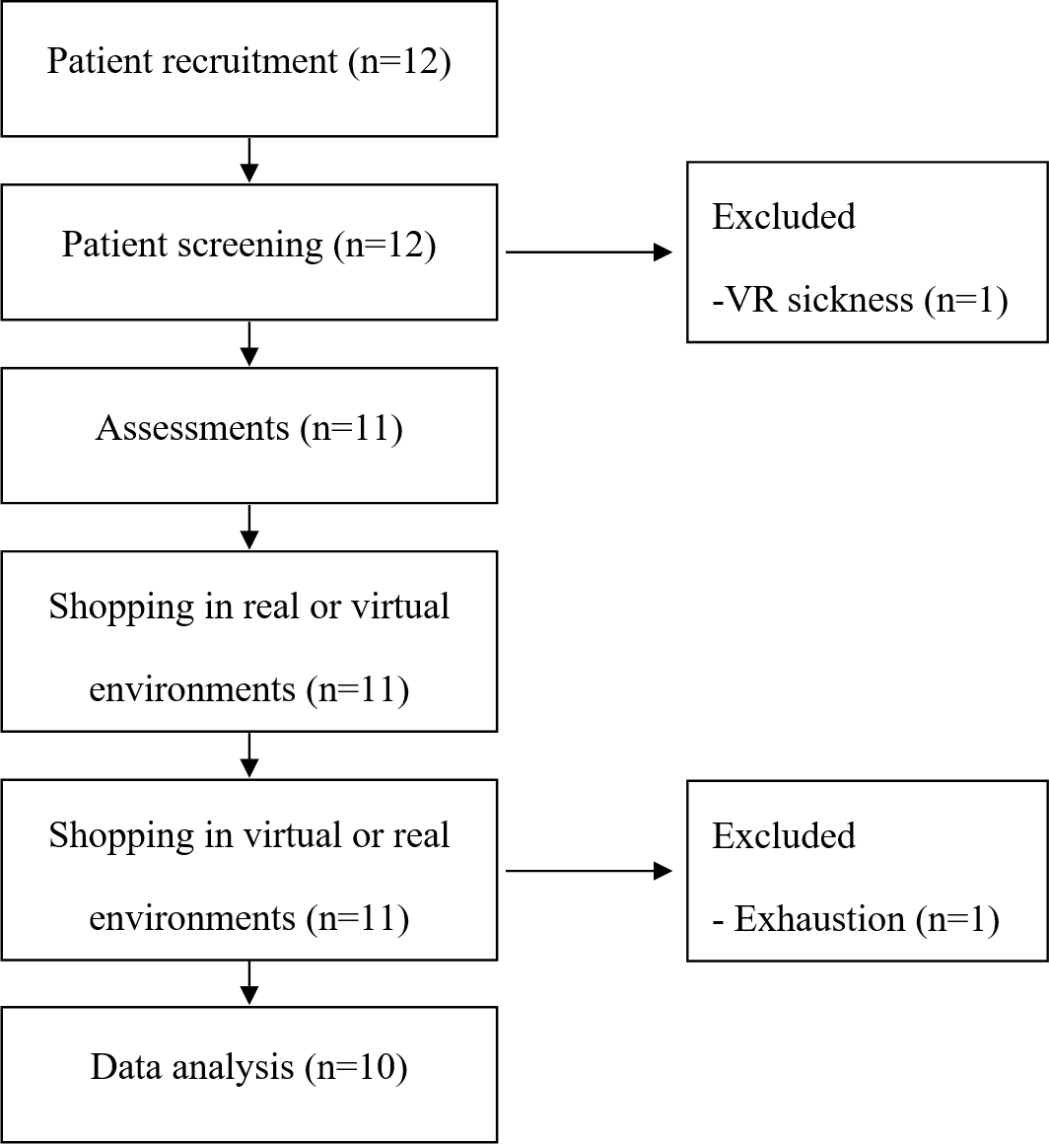
Flowchart of this study.

This pilot study was designed as an observational, randomized, crossover study comparing occupational performance in real-world shopping and IVR shopping among people undergoing stroke rehabilitation (Figure 1). The real-world and virtual shopping sequence was randomized by flipping a coin (heads: real-world shopping first; tails: virtual shopping first). All patients were recruited at the Department of Rehabilitation Medicine, Shenzhen Hospital, Southern Medical University. The rehabilitation physicians screened inpatients with stroke admitted to rehabilitation wards according to the inclusion and exclusion criteria. Once the patients agreed to participate in this study, they were referred to occupational therapists for assessment and implementation of VR training.

### Ethics Approval

This study was approved by the ethics committee of Shenzhen Hospital, Southern Medical University (AF/SC-09/01.0). We adhered to the Declaration of Helsinki of 1975 and 2000 throughout the study, and followed the institutional and national ethical standards on human experimentation. All the patients with stroke in the rehabilitation ward who met the requirements had the right to participate in this study freely, and they could leave the study any time they wanted without any repercussions. This study’s Chinese Clinical Trial Registry registration number is ChiCTR2000041058.

### Inclusion and Exclusion Criteria

All patients were required to meet the following criteria: (1) they had completed primary education (reading was needed throughout the tasks in the virtual and real environments); (2) they had medically stable stroke (defined as having reported no further decline in body function); (3) they had a Brunnstrom stage from 3 to 6; (4) they could ambulate independently with or without assistive devices; (5) they had a Mini-Mental State Examination (MMSE) score of 24 or higher; and (6) they could understand and follow study instructions. Patients were excluded if they met the following criteria: (1) they had any discomfort or contraindications related to the study; (2) they were unable to sign the informed consent form; and (3) they were participating in any other ongoing study.

### Outcome Measures

Shopping accuracy, which indicates recall ability, and time, which reflects executive functioning, are the most important parameters in occupational performance for this task [24]. Regarding the objectives of this study, we aimed to compare performance in virtual and real environments but not to examine the effect of training, and as time performance (ie, executive functioning) can be influenced by multiple confounders, such as recall, ambulation, and upper extremity function, and can lead to observations of less stable performance, we extracted time (ie, duration) for the most stable, best performance of recall (ie, memory capacity). In addition to performance in the virtual and real environments, including memory capacity and duration of shopping, we also used 4 conventional assessments. (1) MMSE—this test is widely used to assess cognitive function in individuals with suspected cognitive impairment. It includes questions and tasks designed to test orientation, attention, memory, language, and visual-spatial skills. The maximum score is 30 points, with higher scores indicating better cognitive function [25]. (2) Timed Up and Go Test (TUGT)—this test assesses ambulation. It measures the time an individual takes to rise from a chair, walk a distance of 3 meters, turn around, walk back to the chair, and sit down again. The test assesses static and dynamic balance, lower extremity strength, and mobility. The best time in 3 attempts is recorded [26]. (3) Modified Barthel index (MBI)—this was used to investigate basic activities of daily living (BADLs). The MBI is a validated scale that measures the ability to perform BADLs such as feeding, bathing, dressing, and grooming. It ranges from 0 (total dependence) to 100 (total independence) [27]. (4) the LI—this index assesses IADLs, including the ability to perform complex daily tasks such as using the telephone, shopping, cooking, and handling finances. It ranges from 0 (total dependence) to 18 (total independence) [28].

An experienced occupational therapist was responsible for conducting all assessments. The patients were assessed after being referred by rehabilitation physicians in charge of screening. The IVR system automatically recorded data related to the virtual environments, while occupational performance in real environments was recorded manually with a stopwatch and a notebook. We followed the instructions described in the following studies: the Chinese version of MMSE, which was investigated by Katzman and colleagues [25], the TUGT, which was reported by Mathias and colleagues [26], the Chinese version of the MBI, which was translated by Leung and colleagues [27], and the Chinese version of the LI, which was described by Tong and Man [28].

### Implementation of the Shopping Tasks

A research student in occupational therapy was in charge of implementing this study. On the second day after assessment, all patients were required to complete shopping tasks in virtual and real environments in one day, with sufficient time (one day before shopping) to explore both environments and eliminate time wasted due to unfamiliar circumstances. To minimize any potential learning effect on occupational performance, the research student confirmed the randomization of the sequence before implementing the study by flipping a coin. The shopping tasks were repeated until the best result for memory capacity was obtained.

**Figure 2.**
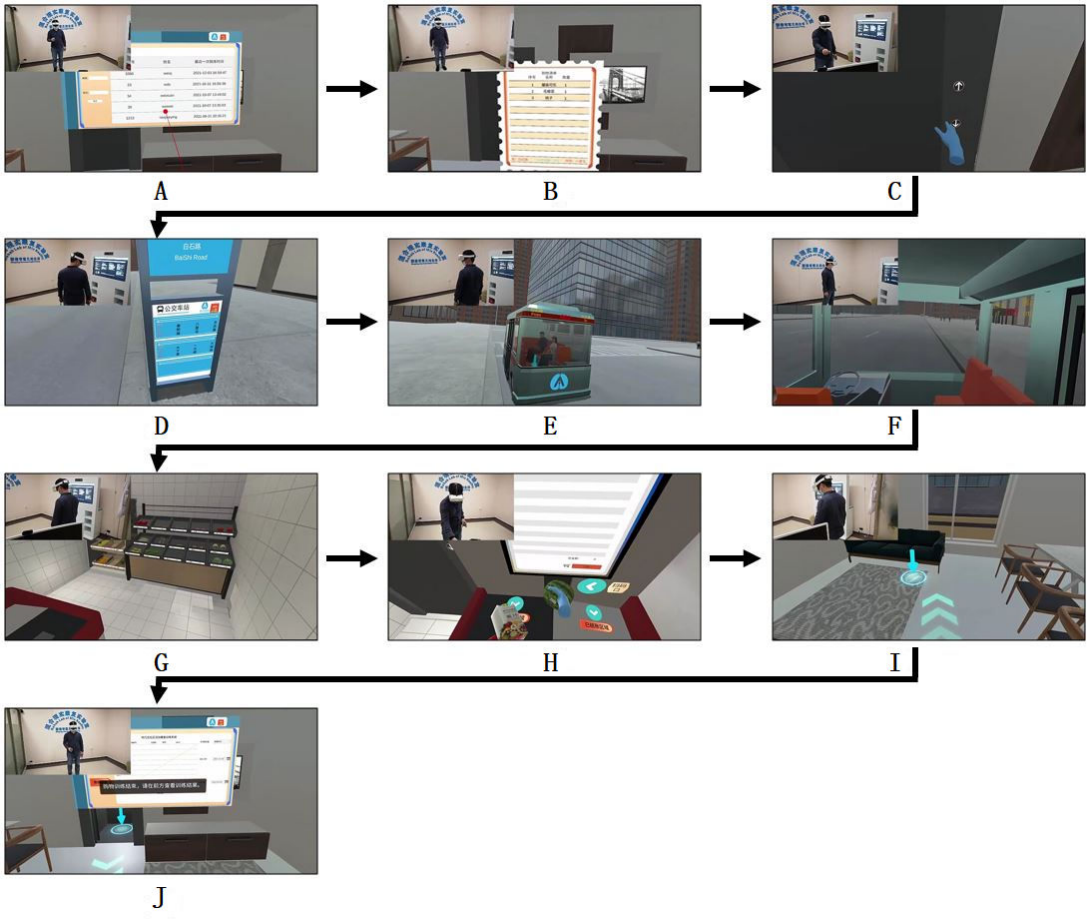
The first author (XW) demonstrates the shopping tasks in the IVR shopping training system: (A) selecting an account, (B) reading the shopping list, (C) entering the elevator, (D) reading the bus stop information board, (E) waiting for the bus, (F) riding the bus, (G) entering the store, (H) settling the payment, (I) returning home, and (J) viewing the results.

The shopping tasks in the virtual environments were delivered using an all-in-one commercially available immersive head-mounted display (PICO Neo 2 Pro; PICO Immersive Pte). This device provides 6 degrees of freedom and enables users to navigate a virtual environment of approximately 9 square meters. All the shopping tasks were integrated into this area. Interactions between patients and the virtual environment, such as picking up goods, pressing buttons, and making payments, rely on holding joysticks in both hands. Figure 2 shows photographs of the display being used and screenshots of the virtual environments. Virtual shopping incorporated several modules, including a virtual home, elevator, bus, and store. After patients entered the system and set parameters, tasks were arranged randomly in the virtual home. Patients had 1 minute to memorize tasks, including a bus route, locations in the home, a bus stop, and a store, as well as the goods they needed to purchase (Figure 2A and B). All patients began by purchasing 4 items, and the number of items (representing memory capacity) increased or decreased automatically to the maximum or minimum for which patients could complete tasks without any mistakes. Virtual shopping could be repeated several times to accurately determine memory capacity. Patients were required to proceed to an elevator, press a button, enter the elevator, exit the elevator, and proceed to a bus stop (Figure 2C-E). The store could be reached by taking the correct bus route and getting off at the correct bus stop (Figure 2F and G). Patients could return home by the reverse route after purchasing their goods and making a payment (Figure 2H and I). Occupational performance throughout the shopping experience was displayed after the patients arrived home (Figure 2J). The research student stood beside the patients during virtual shopping for safety and fall prevention.

We also conducted a real-environment simulation of shopping at our hospital. The patients initiated the shopping tasks in our rehabilitation laboratory on the second floor, which was equipped with VR technology. They then took the elevator to the hospital store on the first floor, as they did in the IVR shopping system. The distance between the laboratory and the store was approximately 120 meters, and the time it took for a healthy adult to traverse this distance was similar to the time it took in the virtual environment. The real store had numerous shelves of goods, and we used 2 shelves specifically for this study. The number of items and strategies for increasing and decreasing the number of items were the same as in the virtual shopping system, but the types of items differed.

### Data Analysis

Nonparametric approaches were used for data analysis because of the small sample size in this study. Comparisons between the virtual and real environments were analysed using the Wilcoxon test, and the correlations between assessments and shopping performance were evaluated using Spearman correlation coefficients (ρ). The hypothesis of this study was that occupational performance in both virtual and real environments would be similar. We used Cohen *d* to estimate the effect size when memory capacity and duration were compared between the virtual and real environments. The Spearman correlation coefficient was used for the effect sizes of correlations between memory capacity, duration, and assessments. The ρ values were interpreted as follows: 0 to 0.25 indicated a weak correlation; 0.25 to 0.5 indicated a fair correlation; 0.50 to 0.75 indicated a moderate correlation; and 0.75 or above indicated an excellent correlation [29]. Assuming a correlation coefficient of 0.8 between the virtual and real environments for memory capacity, the required sample size for this study to detect a significant correlation (*P*<.05) with a 2-tailed test was determined to be 9 according to the sample size table for correlation analysis recommended by Portney and Watkins [29]. CIs are reported in the correlation analysis. The occupational therapist (XW) reviewed any adverse events among the recruited patients during the study. Once an event caused by the VR system such as headache, sickness, exhaustion, or any other discomfort was confirmed, the patient was advised to stop the study.

## Results

Twelve potential patients were recruited from January 3 to June 31 in 2022. Ten of them completed all tasks, while 2 were excluded due to sickness during virtual shopping (n=1) and exhaustion during real-world shopping (n=1) (Figure 1). According to the report from the sick patient, she experienced sickness even when watching conventional video games on a television. These 2 patients stopped the study voluntarily. The recruitment was stopped because the proposed sample size was met.

The demographic characteristics of the recruited patients are summarized in Table 1. The majority of patients were male (n=7, 70%) and had experienced ischemic stroke (n=9, 90%). Their ages ranged from 14 to 73 years, and the time since stroke ranged from 1 to 42 months (n=7, 70% were in the chronic stage).

The Wilcoxon test showed no significant differences in memory capacity and duration between the virtual and real environments (*P*>.99 and *P*=.99). The median values for memory capacity (ie, the maximum numbers of objects a patient could recall, such as fruits, soft drink, and vegetables) were the same in both environments at 3.50 (IQR 3.00-4.75), with a difference of 0. The median values for duration in the virtual and real environments were 604.50 (IQR 549.00-636.75) seconds and 582.50 (IQR 515.50-611.50) seconds, respectively, with a median difference of 21.00 (IQR −98.50 to 72.50). Cohen *d* showed the effect sizes of memory capacity and duration were 0 and 0.02, respectively (Table 2).

The Spearman correlation coefficient showed a significant relationship between memory capacity and the LI in the virtual and real environments (ρ=0.81, 95% CI 0.42‐0.96; *P*=.005). Duration had a moderate relationship with the TUGT in the virtual environment (ρ=0.68, 95% CI 0.13‐0.94; *P*=.03) and a borderline negative correlation with the MMSE in the real environment (ρ=−0.58, 95% CI −0.92 to 0.03; *P*=.08) (Table 3).

**Table 1.** Demographic characteristics of the recruited patients (n=10).

Patient	Gender	Age (years)	Occupation	Education	Type of stroke	Time since stroke (months)	MMSE^a^ score	TUGT^b^ score(seconds)	MBI^c^ value	LI^d^ value
1	Male	59	Retired businessman	High school	Ischemic	7	29	38	100	15
2	Male	50	Businessman	Middle school	Ischemic	9	27	8	100	15
3	Male	66	Retired engineer	University	Hemorrhagic	42	30	57	93	15
4	Female	37	Clerk	High school	Ischemic	8	28	31	86	12
5	Male	38	Businessman	Primary school	Ischemic	3	29	21	100	15
6	Male	14	Student	Middle school	Ischemic	10	30	11	100	16
7	Male	44	Manager	Middle school	Ischemic	4	29	19	100	17
8	Female	73	Farmer	Primary school	Ischemic	1	30	14	100	15
9	Female	30	Unemployed	Middle school	Ischemic	11	25	31	100	10
10	Male	53	Clerk	University	Ischemic	11	29	23	100	18

a MMSE: Mini-Mental State Examination; scored out of 30.

b TUGT: Timed Up and Go Test.

c MBI: modified Barthel index; scored out of 100.

d LI: Lawton index; scored out of 18.

**Table 2.** Occupational performance comparison between the virtual and real environments (n=10).

Occupational performance	Virtual, mean (IQR)	Real, mean (IQR)	*P* value (Wilcoxon test)	Cohen *d*	Difference between virtual and real, mean (IQR)
Memory capacity	3.50 (3.00-4.75)	3.50 (3.00-4.75)	>.99	0	0 (0 to 0)
Duration (seconds)	604.50 (549.00-636.75)	582.50 (515.50-611.50)	.99	0.02	21.00 (−98.50 to 72.50)

**Table 3.** Correlations between occupational performance and demographic characteristics (2-tailed Spearman correlation coefficients with 95% CI; n=10).

Occupational performance		MMSE^a^, ρ (95% CI)	*P* value	TUGT^b^, ρ (95% CI)	*P* value	LI^c^, ρ (95% CI)	*P* value
**Virtual environment**							
	Memory capacity	0.53 (−0.33 to 0.98)	.11	−0.15 (−0.86 to 0.62)	.69	0.81 (0.42 to 0.96)	.005
	Duration (seconds)	−0.41 (−0.95 to 0.39)	.24	0.68 (0.13 to 0.94)	.03	−0.52 (−0.96 to 0.25)	.12
**Real environment**							
	Memory capacity	0.53 (−0.33 to 0.98)	.11	−0.15 (−0.86 to 0.62)	.69	0.81 (0.42 to 0.96)	.005
	Duration (seconds)	−0.58 (−0.92 to 0.03)	.08	0.30 (−0.45 to 0.77)	.40	−0.36 (−0.95 to 0.48)	.31

a MMSE: Mini-Mental State Examination.

b TUGT: Timed Up and Go Test.

c LI: Lawton index.

## Discussion

### Principal Findings

The goal of this research was to investigate the correlation between occupational performance among patients with stroke in shopping tasks in 2 different environments: IVR and the real world. The primary parameters we concentrated on were memory capacity and the time taken (ie, duration) for shopping tasks. Our initial findings preliminarily support our hypothesis that occupational performance in these 2 situations is similar in terms of memory capacity. The findings indicate that IVR shopping could be an alternative assessment and training approach for IADLs.

Exploring the consistency of performance in IVR and real environments is crucial for developing this new technology for rehabilitation. Our preliminary data show no significant difference between IVR and real environments regarding cognition (memory capacity) and movement (duration). Currently, there is limited evidence comparing IVR and real environments, but a systematic review conducted by Tuena and colleagues [20] showed that spatial memory performance was similar in nonimmersive virtual and real environments. A recent review by Palombi and colleagues [30] on IVR and a real radial arm maze found that IVR was suitable for navigation training and promoting spatial memory performance. These findings suggest that cognitive performance in IVR may be comparable to real environments. However, we need to know the difference between IVR and previous technologies. IVR delivers a more immersive environment than nonimmersive technologies and presents virtual tasks that are closer to activities of daily living than a radial arm maze.

Further research is needed to compare movement performance between IVR and real environments. One study on obstacle avoidance distance conducted by Khenak and colleagues [31] showed that movement in a real environment was significantly different from IVR, with a larger avoidance distance observed in a real environment. This finding is partially supported by a study done by Brock and colleagues [18], which found that motor control (movement kinematic and postural control) in golf putting could be influenced by somatosensory input, and better occupational performance could be observed in real environments. However, these studies’ findings should be compared cautiously with ours for several reasons. First, the patients recruited were different in these past studies and our study. We recruited patients who had experienced stroke, whereas the 2 previous studies recruited healthy adults and undergraduate students. Impaired ambulation could be a critical factor that influences movement performance and masks the contribution of differences between environments. Second, different systems were used and different parameters were extracted. We simulated a full route of community shopping based on usual activities of daily living–related training in conventional rehabilitation and extracted duration as the movement performance. Still, the 2 previous studies used indoor navigation and golf putting, and the movement patterns of these activities were simpler and shorter aspects of activities of daily living. Finally, the sample size could be an important element in reducing the power of this study. These findings might suggest that the consistency of movement performance in virtual environments compared to real environments presents variable outcomes that further influence occupational performance.

IADLs refer to the integration and performance of body functions, that is, cognition and movement [3]. Our study found a positive correlation between cognition (memory capacity) and IADLs (the LI), with a strong correlation coefficient of 0.81. This correlation was consistent across both IVR and real environments. This result is partially supported by a study conducted by Ghaffari and colleagues [32], which found that memory, as assessed with the Wechsler memory scale, correlated moderately with the LI. However, our study did not find a significant correlation between movement (duration) and the LI. A previous study reported a moderate correlation between movement performance as assessed by the Motricity index for the upper and lower extremities and the LI [32]. The varying movement performance observed may have led to this conflict. Multiple tasks could have influenced the duration of virtual ambulation throughout such activities of daily living as shopping in this study. At the same time, fewer confounders affected muscle strength, and the previous study used fewer activities of daily living. Currently, the relationship between movement and IADLs in virtual environments has not been extensively studied. Our findings suggest that cognitive performance is comparable in virtual and real environments, but the consistency of movement performance could be variable due to differences in confounders.

Interestingly, our results indicated a moderate and significant correlation between duration and the TUGT in the IVR environment (ρ=0.68; *P*=.03), but this correlation was not significant in the real-world environment. However, a borderline moderate negative correlation was observed between the duration and MMSE scores in the real environment (ρ=−0.58; *P*=.08), with no significance seen in IVR. The TUGT and MMSE are valid and reliable assessments for ambulation and cognition screening, respectively [25,26]. These results suggest that movement performance (duration) in a virtual environment may predict the ability to ambulate (but not in the real environment). Conversely, cognition (MMSE) is a critical factor influencing movement performance in real environments (but not virtual ones). These conflicts might be attributed to the varying experiences encountered in virtual and real environments, such as the reduced somatosensory input in a virtual environment [18,19].

### Limitations

The limitations of this study include the following: (1) The small sample size met the requirement of memory capacity but not other parameters and assessments, which could have influenced the power of this preliminary study. We suggest increasing the sample size according to the research topic. (2) A public bus route was not used in the real environment, which was a prominent difference between IVR and the real environment. This difference might have influenced shopping performance in the virtual and real environments. Replacing bus-taking with walking in the virtual environment or adding a simulation of bus-taking to the real environment are possible solutions for this. (3) We recruited patients who had experienced stroke who had good functioning and literacy to guarantee compliance and executive function. Patients with low functioning or who were illiterate might have presented different results, so the representativeness of this study could have been influenced. Slightly increasing the variety of recruited patients could enhance the generalizability of the findings. (4) Memory capacity and duration are only partial parameters; they cannot show the full picture of occupational performance. Exploring more sensitive parameters and assessments according to the research focus could be helpful. Readers should be cautious when interpreting the findings of this study.

### Future Directions

Future research could proceed in the following directions: (1) increasing the sample size, which could lead to more findings; (2) using different types of human-computer interaction and data extraction, which could influence, respectively, occupational performance and observations of different dimensions of occupational performance; (3) extending the use of IVR to patients with stroke who have worse functioning; (4) exploring the effect of training and the transfer of gains in IVR to the real environment; and (5) assessing the benefits of IVR in different circumstances, such as home-based and community-based rehabilitation and telerehabilitation.

### Conclusions

In conclusion, considering the small sample size used in this study and the study’s limitations, despite the significant correlation between shopping performance in IVR and the real world, it is still too early to conclude that IVR is a noninferior approach, although it has the potential to be an alternative approach for assessment and training for IADLs when resources are limited. However, further research is needed to investigate the psychometric properties of this novel assessment, the effectiveness of training in IVR, and whether the effect can be translated to the real-world environment. The implications for practice might include the following: (1) occupational performance in virtual shopping might be the same as real-world shopping, and more virtual IADLs could be developed, especially for activities that are challenging in real environments; (2) virtual IADL assessment and training systems could be used in remote locations or limited-resource settings, such as community-based rehabilitation and telerehabilitation; (3) more objective parameters of IADLs could be extracted from virtual environments, such as balance, ambulation, and cognition.

## Abbreviations

BADL: basic activity of daily living
IADL: instrumental activity of daily living
*ICF*: *International Classification of Functioning, Disability and Health*
IVR: immersive virtual reality
LI: Lawton index
MBI: modified Barthel index
MCI: mild cognitive impairment
MMSE: Mini-Mental State Examination
TUGT: Timed Up and Go Test
VR: virtual reality
